# Effects of a single intraperitoneal administration of cadmium on femoral bone structure in male rats

**DOI:** 10.1186/1751-0147-53-49

**Published:** 2011-08-31

**Authors:** Monika Martiniaková, Hana Chovancová, Radoslav Omelka, Birgit Grosskopf, Róbert Toman

**Affiliations:** 1 Department of Zoology and Anthropology, Constantine the Philosopher University, Nábrežie mládeže 91, 949 74 Nitra, Slovak Republic; 2 Department of Botany and Genetics, Constantine the Philosopher University, Nábrežie mládeže 91, 949 74 Nitra, Slovak Republic; 3 Johann Friedrich Blumenbach Institute of Zoology and Anthropology, Georg-August University, Bürgerstrasse 50, 37 073 Göttingen, Germany; 4 Department of Veterinary Sciences, Slovak University of Agriculture, Trieda Andreja Hlinku 2, 949 76 Nitra, Slovak Republic

## Abstract

**Background:**

Exposure to cadmium (Cd) is considered a risk factor for various bone diseases in humans and experimental animals. This study investigated the acute effects of Cd on femoral bone structure of adult male rats after a single intraperitoneal administration.

**Methods:**

Ten 4-month-old male Wistar rats were injected intraperitoneally with a single dose of 2 mg CdCl_2_/kg body weight and killed 36 h after the Cd had been injected. Ten 4-month-old males served as a control group. Differences in body weight, femoral weight, femoral length and histological structure of the femur were evaluated between the two groups of rats. The unpaired Student's t-test was used for establishment of statistical significance.

**Results:**

A single intraperitoneal administration of Cd had no significant effect on the body weight, femoral weight or femoral length. On the other hand, histological changes were significant. Rats exposed to Cd had significantly higher values of area, perimeter, maximum and minimum diameters of the primary osteons' vascular canals and Haversian canals. In contrast, a significant decrease in all variables of the secondary osteons was observed in these rats.

**Conclusions:**

The results indicate that, as expected, a single intraperitoneal administration of 2 mg CdCl_2_/kg body weight had no impact on macroscopic structure of rat's femora; however, it affected the size of vascular canals of primary osteons, Haversian canals, and secondary osteons.

## Background

Cadmium (Cd) is considered a dangerous poison for humans and animals. Adverse health effects caused by accidental, spontaneous or experimental exposure to Cd have led to significant public health concerns [1]. Depending on the dose, route and duration of exposure, Cd can damage various organs [2]. The kidneys, liver, bones, and respiratory and cardiovascular systems are the most important target organs for Cd toxicity [3].

Changes in bone such as osteopenia, osteoporosis, and osteomalacia, with increased bone fragility and pathological fractures have been noted in humans and experimental animals as a result of exposure to Cd [4-9]. In general, bone toxicity of Cd can be modulated by both direct and indirect mechanisms [10,11]. The direct action is associated with altered osteoblastic and osteoclastic activities [12] resulting in decreased bone formation [13,14] and enhanced bone resorption [15,16]. The indirect mechanism involves abnormal metabolism of vitamin D and minerals, mainly calcium (Ca), due to kidney and gastrointestinal tract damages [17,18]. Besides interfering with Ca metabolism, Cd also alters the metabolism of other metals essential for bone health, mainly zinc (Zn), iron (Fe) and copper (Cu) [19]. Bonner *et al. *[20] have reported a decreased concentration of both Ca and Zn in the femur of rats after a single injection of CdCl_2_. However, there have been no reports on changes in bone structure after acute exposure to Cd.

Therefore, the aim of the present study was to determine if there are acute effects of Cd on femoral bone structure in adult male rats when Cd is administered intraperitoneally in a single dose.

## Methods

### Animals

Twenty clinically healthy 4-month-old male Wistar rats (Slovak University of Agriculture, Nitra, Slovak Republic), were divided randomly into two groups of ten animals each. Male rats were used as they are less susceptible than females. The rats were housed individually in plastic cages in an environment maintained at 20-24°C, 55 ± 10% humidity. They had access to water and food (feed mixture M3, Bonagro, Czech Republic) *ad libitum*. Rats of group 1 were injected intraperitoneally with a single dose of 2 mg CdCl_2_/kg body weight (BW) (Reachem, Slovak Republic) and were killed after 36 h. Ten 4-month-old males (group 2) served as an untreated control group and were kept and killed parallel to the Cd exposed animals. The rats were kept for other investigations (e.g., histological and biochemical analyses) at the Slovak University of Agriculture. In these investigations the acute intraperitoneal administration of Cd in one dose is generally applied to reveal possible manifestations of Cd intoxication in different tissues. The present study was performed as an additional investigation focused on bone tissue. All procedures were approved by the Animal Experimental Committee of the Slovak Republic.

### Procedures

At the end of the experiment, all animals were euthanized and weighed. The right femur (n = 20) was sampled at necropsy, weighed and measured using analytical scales and a sliding instrument. Specimens were sectioned at the diaphyseal midshaft. Two transversal sections were taken from each femur. The obtained segments were placed in HistoChoice fixative (Amresco, USA). Specimens were then dehydrated in ascending grades of ethanol and embedded in epoxy resin Biodur (Günter von Hagens, Heidelberg, Germany) according to Martiniaková *et al. *[21]. Histological sections (70-80 μm thick) were prepared with a sawing microtome (Leitz 1600, Leica, Wetzlar, Germany) and affixed to glass slides by Eukitt (Merck, Darmstadt, Germany) as previously described [22]. The qualitative histological characteristics were determined according to the classification systems by Enlow and Brown [23] and Ricqlès *et al. *[24]. The quantitative variables were assessed using the software Motic Images Plus 2.0 ML (Motic China Group Co., Ltd.) in anterior, posterior, medial and lateral views of thin sections. At least 20 vascular canals of primary osteons were measured in each individual (5 each of anterior, posterior, medial and lateral views). All secondary osteons, which were not in a resorption phase and could clearly be outlined using the Motic Images Plus 2.0 ML software were measured. Magnification of 225 times was used for histomorphometrical measurements. Secondary osteons were distinguished from primary osteons (i.e., primary vascular canals) on the basis of a well defined peripheral boundary (cement line) between the osteon and the surrounding tissue.

### Statistics

Statistical analysis was performed using SPSS 8.0 software. All data were expressed as mean ± standard deviation (SD). The unpaired Student's t-test was used for establishing statistical significance (significance level of *P *< 0.05) between experimental and control groups.

## Results

Body weight, femoral weight and length did not differ significantly between groups (Table 1).

**Table 1:** Average body weight, femoral weight and femoral length in rats injected intraperitoneally with a single dose of 2 mg CdCl_2_/kg body weight (group 1) and controls (group 2)

	*N*	*Body weight* *(g)*	*Bone weight* *(g)*	*Bone length* *(cm)*
Group 1	10	430 ± 55.33	1.12 ± 0.16	3.95 ± 0.21
Group 2	10	405 ± 52.65	1.05 ± 0.17	3.94 ± 0.09
T-test		NS	NS	NS

N: number of rats, NS: non-significant changes

**Figure 1:**
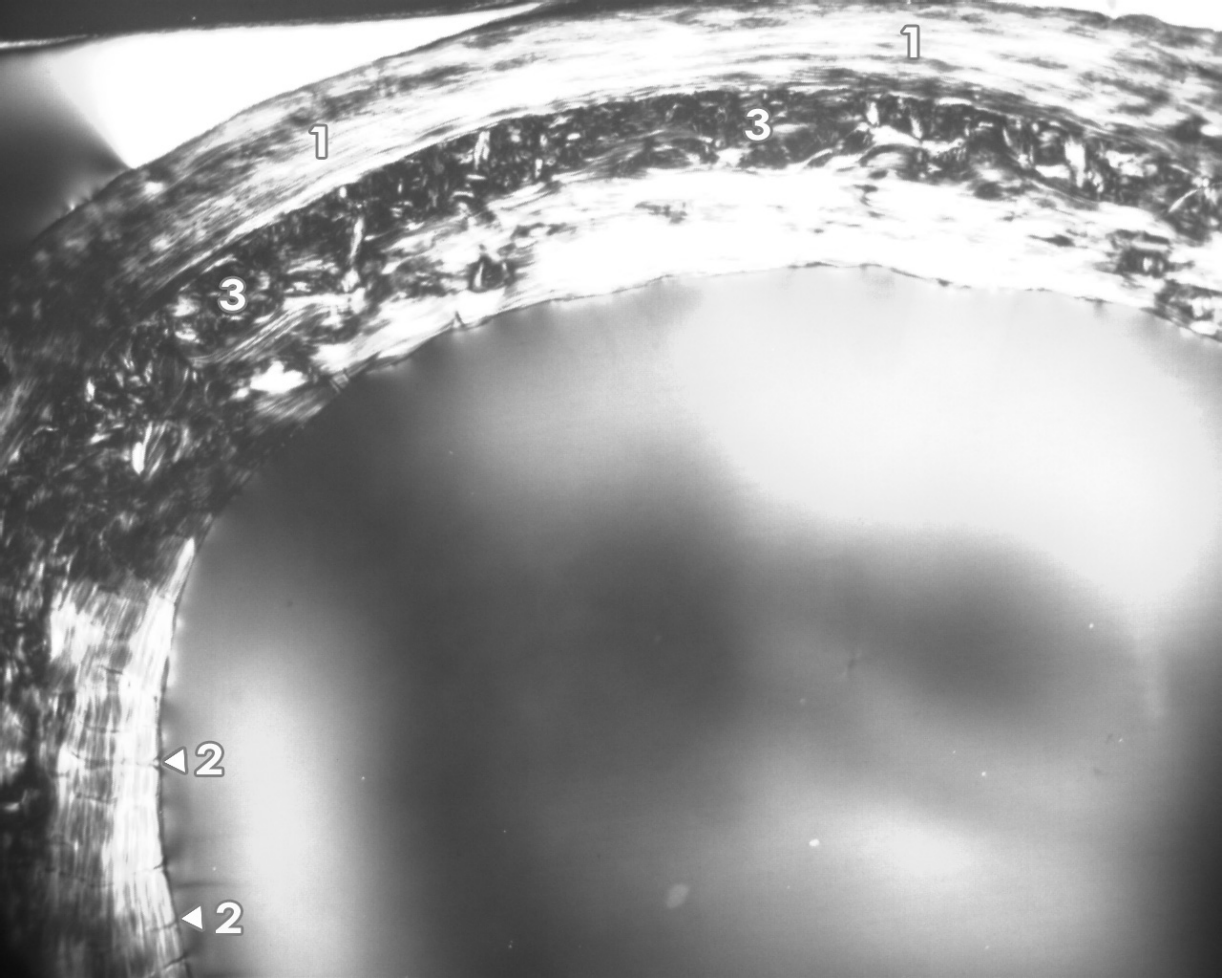
**Microscopic structure of femoral bone tissue in rats injected intraperitoneally with a single dose of 2 mg CdCl_2_/kg body weight**. 1 - non-vascular bone tissue. 2 - vascular canals radiating from marrow cavity. 3 - primary and secondary osteons in middle part of substantia compacta.

**Figure 2:**
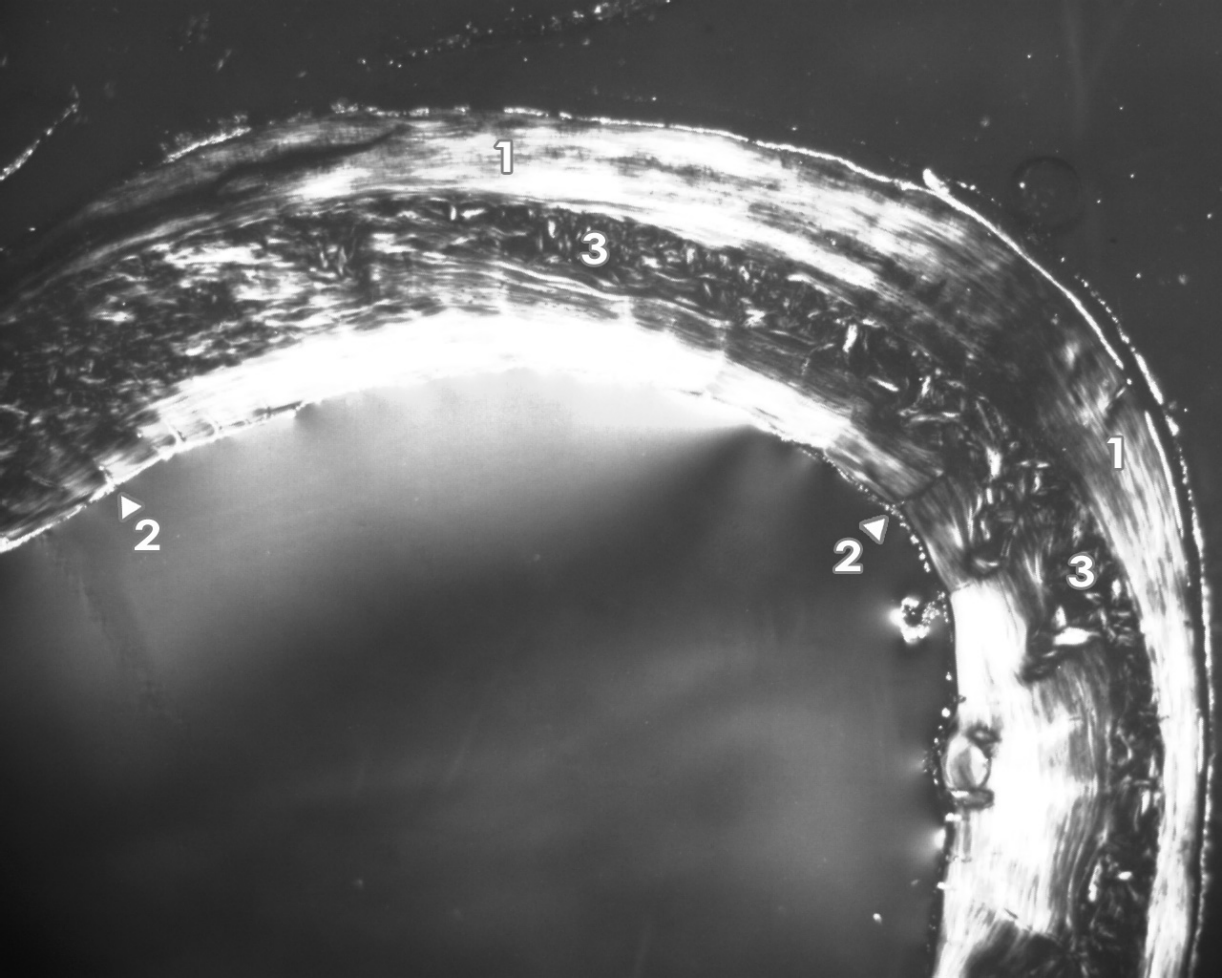
**Microscopic structure of femoral bone tissue in rats from the control group**. 1 - non-vascular bone tissue. 2 - vascular canals radiating from marrow cavity. 3 - primary and secondary osteons in middle part of substantia compacta.

The femora of all rats had the following microstructure in common. An endosteal border was formed by non-vascular bone tissue and/or primary vascular radial bone tissue. Non-vascular bone tissue contained cellular lamellae and osteocytes. Primary and secondary osteons were absent. Primary vascular radial bone tissue created branching or non-branching vascular canals radiating from the marrow cavity. Some primary and secondary osteons were exceptionally found in anterior and posterior views near the endosteal surfaces. In the middle parts of the compact bone, a few primary and secondary osteons were identified. However, dense Haversian bone tissue characterized by dense concentration of secondary osteons was not observed in rats from either group. Finally, the periosteal border was composed of non-vascular bone tissue, mainly seen in anterior and posterior views (Figures 1 and 2). In general, significant differences in qualitative histological characteristics of the compact bone tissue between groups were not observed. No resorption lacunae and/or osteoporotic fractures were found.

Quantitative observations, on the other hand, did differ between the two groups. Data on bone measurements are shown in Tables 2, 3 and 4. The values for the vascular canals of primary osteons were higher in Cd exposed rats. Significant differences were found for area, perimeter, maximum and minimum diameters of primary osteons' vascular canals, i.e. all measured variables. Higher values for all variables of the Haversian canals were also recorded in the rats exposed to Cd (*P *< 0.05). On the other hand, all variables of the secondary osteons had higher values in rats from the control group. Statistically significant differences were identified for area, perimeter, maximum and minimum diameters of the secondary osteons.

**Table 2:** Data on vascular canals of primary osteons

	*N*	*Area* *(μm^2^)*	*Perimeter* *(μm)*	*Max.diameter* *(μm)*	*Min.diameter* *(μm)*
Group 1	395	446.26 ± 64.11	76.03 ± 5.92	13.24 ± 1.54	10.79 ± 1.15
Group 2	436	393.49 ± 123.04	71.58 ± 12.37	12.86 ± 2.67	9.62 ± 1.68
T-test		1 *vs*.2*	1 *vs*.2*	1 *vs*.2*	1 *vs*.2*

*P < 0.05, N: number of measured structures

Data on vascular canals of primary osteons in rats injected intraperitoneally with a single dose of 2 mg CdCl_2_/kg body weight (group 1, 10 rats) and controls (group 2, 10 rats).

**Table 3:** Data on Haversian canals

	*N*	*Area* *(μm^2^)*	*Perimeter (μm)*	*Max.diameter* *(μm)*	*Min.diameter* *(μm)*
Group 1	254	468.26 ± 71.44	77.66 ± 6.02	13.43 ± 1.50	11.14 ± 1.15
Group 2	227	428.30 ± 75.75	74.94 ± 7.30	13.04 ± 1.56	10.73 ± 1.18
T-test		1 *vs*.2*	1 *vs*.2*	1 *vs*.2*	1 *vs*.2*

* P < 0.05, N: number of measured structures

Data on Haversian canals in rats injected intraperitoneally with a single dose of 2 mg CdCl_2_/kg body weight (group 1, 10 rats) and controls (group 2, 10 rats).

**Table 4:** Data on secondary osteons

	*N*	*Area* *(μm^2^)*	*Perimeter* *(μm)*	*Max. diameter* *(μm)*	*Min. diameter* *(μm)*
Group 1	254	5284.90 ± 1080.44	260.92 ± 27.02	45.52 ± 5.69	36.85 ± 4.79
Group 2	227	6408.74 ± 1820.98	292.98 ± 39.59	51.64 ± 8.17	39.51 ± 6.20
T-test		1*vs*.2*	1 *vs*.2*	1 *vs*.2*	1 *vs*.2*

* P < 0.05, N: number of measured structures

Data on secondary osteons in rats injected intraperitoneally with a single dose of 2 mg CdCl_2_/kg body weight (group 1, 10 rats) and controls (group 2, 10 rats).

## Discussion

A single intraperitoneal dose has been used to investigate possible acute toxic effects of Cd. The dose was high enough to reach a toxicity level but on the other hand sufficient low to prevent animal mortality. Studies of the Cd effects on bone structure contribute to the knowledge on the mechanisms of Cd in the organism and the relations between changes found in different organs. Generally, toxic effects of Cd on bone tissue are studied mainly in relation to the long-term per oral intake of Cd but it is also interesting to see how Cd affects bone structure and function after an acute intoxication. To date, only a few studies focusing on the effects of Cd intoxication on body weight and tissue quantitative changes, (including bone) have been published. With respect to intraperitoneal administration of a low dose of Cd, Comelekoglu *et al. *[25] found no change in femoral length of adult female rats administered intraperitoneally with 0.5 mg CdCl_2_/kg BW three times a week for 18 weeks. Similar to this, we did not observe a significant effect from a single intermediate dose of Cd on femoral length in adult male rats. Also, body weight and femoral bone weight did not differ statistically between the two groups thus demonstrating that a single intermediate dose of Cd given intraperitoneally has no impact on the macroscopic structure of adult rat's femora.

The results of the qualitative histological analysis correspond to those reported by other researchers working with rats [26-28]. The basic structural pattern of compact bone tissue was non-vascular. In addition, primary vascular radial and/or irregular Haversian bone tissues were observed. However, there was no evidence of true Haversian intracortical bone remodelling. It is generally known that aged rats and mice lack true Haversian cortical bone remodelling but not cancellous bone remodelling [29]. Therefore, some secondary osteons can be observed in long bones near the endosteal border. In our study, the newly formed remodelling units within compact bone originated from the endocortical surface and extended deeply into the underlying compact bone. The same observations were also done by Reim *et al. *[28] for 13-month-old male rats. We found no resorption lacunae and/or osteoporotic fractures in the Cd exposed rats. This confirms that a single intermediate dose of Cd given intraperitoneally does not cause osteoporosis (e.g., a lot of resorption lacunae, osteoporotic fractures) within 36 hours.

Morphometrical measurements showed a significant increase in area, perimeter, maximum and minimum diameters of the primary osteons' vascular canals, and Haversian canals in group 1 rats. The increase in size of vascular canals (including Haversian canals) may be due to demineralization and bone vascularization. It has been demonstrated that Cd-induced demineralization begins soon after exposure, e.g. within 24 hours of an oral administration and even earlier following intraperitoneal administration in mice [7]. A dilatation of Haversian canals has also been observed in mice [30] and rats [31] after intraperitoneal administration of Cd. Our results demonstrate that the dilatation can occur after a single intraperitoneal Cd application. In general, the Haversian canals contain a capillary with a complete endothelial lining, an intact basement membrane, and mesenchymal cells at various stages of differentiation in the extravascular space [32]. Also, it is known that Cd induces hypertrophy of the nucleus and swelling of the cytoplasmatic extension of the endothelial cell [33], which could partially contribute to the increased size of the Haversian canals.

We observed a significant decrease in all variables of the secondary osteons. A reduced size of secondary osteons may be seen in animals with shorter bones [34] but Cd exposure did not affect bone length in our study. Cd may replace Ca in hydroxyapatite crystals [13,35]. Blumenthal *et al. *[13] showed that Cd incorporation into hydroxyapatite introduced little strain in the lattice but resulted in a decreasing C-axis spacing and a corresponding crystal size decrease in the C-axis direction. Hydroxyapatite crystals, as a major mineral component of bones, are aligned with their long axis parallel to the collagen fiber axis [36], creating concentric lamellae of the secondary osteon. Therefore, we speculate that a decreased size of hydroxyapatite crystals could partially contribute to the decreased size of the secondary osteons found in Cd exposed rats.

The mean diameters of primary osteons' vascular canals, Haversian canals and secondary osteons in rats of the control group were 8.44 ± 1.11 μm, 9.46 ± 1.38 μm, and 40.21 ± 6.62 μm, respectively. Using the classifications of Rämsch and Zerndt [37] and Gladuhsew [38] these results show that vascular canals of primary osteons and Haversian canals are very short in 4 month-old male rats. Similar results have also been observed in 6 month-old individuals [27].

## Conclusions

Body weight, femoral weight and femoral length were unaffected in adult male rats following a single intraperitoneal administration of 2 mg CdCl_2_/kg BW. However, changes in femoral bone histomorphometry were significant as Cd exposed rats had higher values for area, perimeter, minimum and maximum diameters of primary osteons' vascular canals and Haversian canals while the similar variables for secondary osteons were lower.

## Competing interests

The authors declare that they have no competing interests.

## Authors' contributions

MM was responsible for histological analysis. HC was responsible for macroscopical analysis and photodocumentation of histological sections. RO was responsible for the statistical analysis. BG was responsible for preparation of histological sections. RT was responsible for animal care and sampling of femora. All authors read and approved the final manuscript.
